# Correlation between Multi-Drug Resistance-Associated Membrane Transport in Clonal Cancer Cells and the Cell Cycle Phase

**DOI:** 10.1371/journal.pone.0041368

**Published:** 2012-07-25

**Authors:** Vasilij Koshkin, Sergey N. Krylov

**Affiliations:** Centre for Research on Biomolecular Interactions, York University, Toronto, Ontario, Canada; University of São Paulo, Brazil

## Abstract

Multidrug resistance driven by ABC membrane transporters is one of the major reasons for treatment failure in human malignancy. Some limited evidence has previously been reported on the cell cycle dependence of ABC transporter expression. However, it has never been demonstrated that the functional activity of these transporters correlates with the cell cycle position. Here, we studied the rate of intrinsic ABC transport in different phases of the cell cycle in cultured MCF-7 breast cancer cells. The rate was characterized in terms of the efflux kinetics from cells loaded with an ABC transporter substrate. As averaging the kinetics over a cell population could lead to errors, we studied kinetics of ABC transport at the single-cell level. We found that the rate of ABC transport in MCF-7 cells could be described by Michaelis-Menten kinetics with two classical parameters, *V*_max_ and *K*_M_. Each of these parameters showed similar unimodal distributions with different positions of maxima for cell subpopulations in the 2c and 4c states. Compared to the 2c cells, the 4c cells exhibited greater *V*_max_ values, indicating a higher *activity* of transport. They also exhibited a greater *V*_max_/*K*_M_ ratio, indicating a higher *efficiency* of transport. Our findings suggest that cell cycle-related modulation of MDR may need to be taken into account when designing chemotherapy regimens which include cytostatic agents.

## Introduction

One of the major problems in cancer treatment is the decreased or abolished tumor response to chemotherapies, which is associated with so-called multidrug resistance (MDR)**^1^**, driven by a superfamily of ATP-binding cassette (ABC) plasma membrane transporters [1]. These ABC transporters perform energy-dependent outward transport of a wide range of xeno- and endo-biotics from cells; in particular, anticancer drugs from cancer cells. Attempts to control MDR in cancer cells have, so far, not produced clinically-appreciable results.

It was previously suggested that multidrug resistance is correlated with a cell's position in the cell cycle [2]. This correlation could be useful for current chemotherapy treatments that combine both cytotoxic and cytostatic agents [3], to allow the preferential accumulation of tumor cells in one or another phase of the cell cycle. Understanding the relation between MDR and the cell cycle is essential to ensure that cells are not prompted into a cytotoxin-resistant phase of the cell cycle.

**Figure 1:**
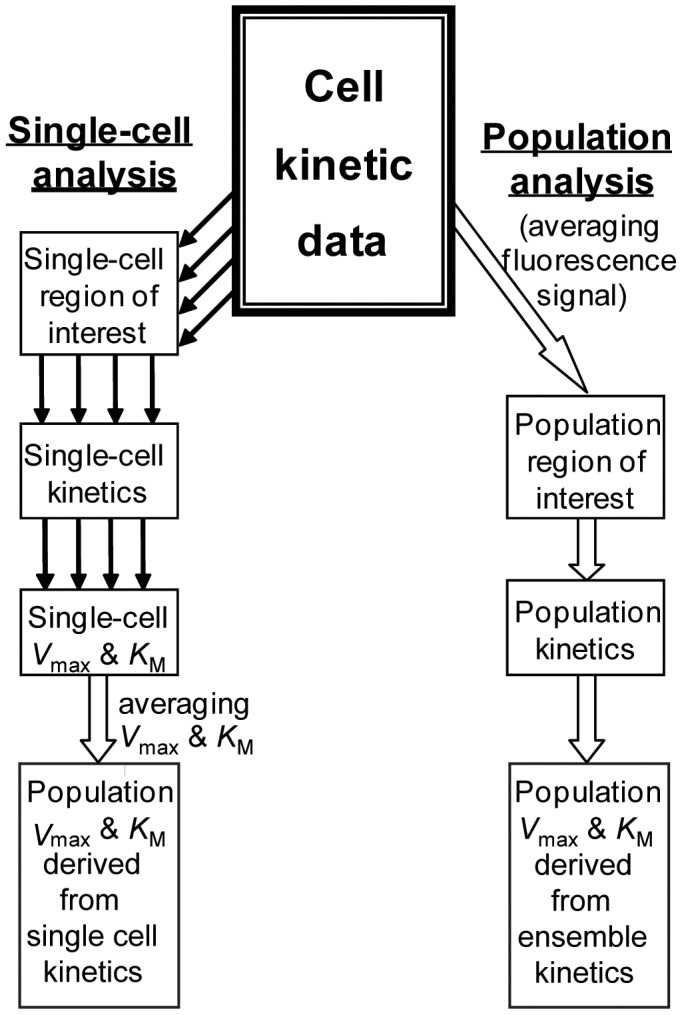
Kinetic analysis of Michaelis processes in cell populations using single-cell and population approaches. Determination of *V*_max_ and *K*_M_ for cell population by averaging: (i) single-cell *V*_max_ and *K*_M_ (single-cell approach) and (ii) single-cell raw signals (population approach).

The current evidence linking cell cycle progression with MDR is fragmentary or incomplete, and presented mostly by correlation data between cell cycle phases and the expression levels of certain ABC transporters [2], [4], [5]. The expression data may not be conclusive, as MDR capacity in cancer cells is determined not only by the expression of plasma membrane transporters of the ABC family, but by a multitude of other factors, including the composition and the fluidic state of plasma membrane, the presence of relevant cofactors, modulators, etc. Thus, the commonly-used assessment of MDR capacity through the analysis of transporter expression was found to be unreliable in a number of cases [6]–[8]. Our work was motivated by the insight that the direct kinetic measurements of MDR transport could serve as an ultimate indicator of cell chemoresistance. We hypothesized that the cells progression through the cell cycle can be accompanied with a modulation in drug efflux kinetics – a net result of cell cycle-related proteomic, membrane, and metabolic changes in the cell. In an effort to reveal subtle intercellular distinctions in MDR activity, we recently developed a single-cell-kinetics approach and demonstrated its high sensitivity for detecting intercellular variation of membrane transport [9]. Here we present a detailed study of cell cycle modulation of MDR transport by using the single-cell-kinetics approach (Fig. 1). It provides functional evidence for cell-cycle-related modulation of MDR activity and suggests that this effect should potentially be considered in the design of combination chemotherapy regimens.

## Materials and Methods

### Chemicals and Materials

MCF-7 cells (human breast cancer cell line [10], [11]) were purchased from the American Type Culture Collection (ATCC, Manassas, VA; ATCC # HTB-22), grown in the recommended media and supplements at 37°C in a humidified 5% CO_2_ environment and used within a 6-month time period. Fluorescent MDR probes rhodamine 123, fluorescein, mithoxantrone, as well as propidium iodide (PI), were purchased from Sigma-Aldrich (St. Louis, MO). All other reagents were obtained from Sigma-Aldrich, Fluka AG Buchs (Switzerland), and BDH Chemicals Ltd. (Poole, England).

### Measurement of Accumulation and Efflux of Fluorescent MDR Probes in Cell Populations by Flow Cytometry

Cellular contents of fluorescent MDR probes were determined by flow cytometry (BD FACSCanto II flow cytometer, BD Biosciences, Franklin Lakes, NJ) in a medium comprised of 140 mM NaCl, 3 mM KCl, 10 mM Na_2_HPO_4_, 2 mM KH_2_PO_4_ 2 mM CaCl_2_, 1 mM MgCl_2_, and 10 mM glucose. After being loaded with the probe (1−5 µM, 30−45 min, 37°C), cells (approximately 10^6^ cells/mL) were sedimented, washed, resuspended in fresh medium (containing 5 µM PI), and examined for intracellular probe content using a standard argon-ion laser emitting at 488-nm for fluorescence excitation and a 530/30 nm band-pass emission filter for the fluorescence detection of rhodamine 123, calcein, and fluorescein and a 585/42 band pass emission filter for the detection of PI. To evaluate the accumulation of mitoxantrone, the samples were excited with a 635-nm red diode laser, and a 680/32 nm band pass emission filter was used to detect fluorescence. For the quantitative kinetic MDR study, the progression of dye efflux was monitored by flow cytometric repetitive sampling of cell suspensions at appropriate time points.

### Measurement of MDR Transport in Single Cells by Fluorescence Kinetic Microscopy

Cells grown to 50–60% confluence were supplemented with 10 µM glyburide (MDR inhibitor) and loaded with 5 µM fluorescein for 30 min at 37°C. Cells were then washed free of extracellular fluorescein and glyburide and placed in the KRB buffer. The kinetics of fluorescein efflux was monitored with 5 min intervals using a laser scanning confocal fluorescence microscope (Fluoview FV300, Olympus, Japan), with an argon-ion laser excitation at 488 nm and the XF75 Omega filter set (Omega Optical, Inc. Brattleboro, VT) following an approach described elsewhere [12].

### Determination of Cell Viability and Cell Cycle Position

After the completion of fluorescein efflux, cells were loaded with PI (10 µM, 10 min) to identify apoptotic cells. The cells were then treated with the plasma membrane-specific detergent, saponin (80 µg/mL) and RNAase A (0.02 mg/mL) to allow for PI intercalation with nuclear DNA. Fluorescence from the PI-treated cells was excited with a green Kr laser and detected with the XF35 Omega filter set (Omega Optical, Inc. Brattleboro, VT). Fluorescence intensity of PI was used to determine cell position in the cell cycle [12]–[14].

### Kinetic Fitting and Simulation Methods

Kinetics of MDR transport was described by progress curves of fluorescein efflux which were fitted to the integrated Michaelis-Menten equation for a single-substrate irreversible reaction:
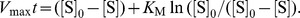
where [S]_0_ and [S] are the initial and current substrate concentrations, respectively [15], [16]. This equation was also used for building the population kinetics models. Fitting and model simulations were carried out with KaleidaGraph (Synergy Software, Reading, PA) and Origin (Microcal Software, Northampton, MA) software.

## Results

### General Characterization of Intrinsic Multidrug Resistance in MCF-7 Cells

**Figure 2:**
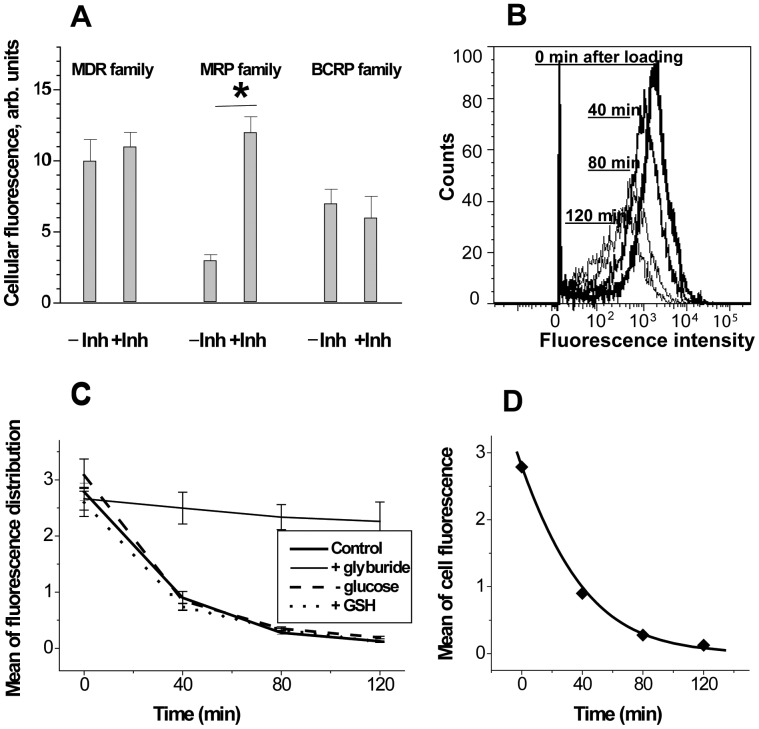
Characterization of intrinsic MDR in MCF-7 cell populations. ***A***, presence of intrinsic MDR activities in MCF-7 cells was estimated by accumulation of: rhodamine 123 without or with cyclosporine A (−/+inh) – for MDR family of transporters; fluorescein without or with glyburide - for MRP family; mithoxantrone without or with cyclosporine A - for BCRP family. ***B***, representative sequence of flow cytometric histograms, in the course of fluorescein efflux, acquired 0, 30, 60 and 90 min after loading. ***C***, kinetics of fluorescein efflux, effect of glyburide application (inhibitor of MRP-type transporters), glucose withdrawal, and application of ethyl ester of GSH. ***D***, Michaelis fit to the progress of fluorescein efflux in cell population.

Initial study on the intrinsic chemoresistance of MCF-7 cells was performed using flow cytometric measurements of MDR substrate accumulation in cells and its dependence on MDR inhibitors. Cells were loaded with combinations of classically-used fluorescent substrates (rhodamine 123, mitoxantrone, and fluorescein) and inhibitors (cyclosporine A, verapamil, and glyburide) specific for distinct families of MDR transporters [17], [18]. These measurements showed that only the fluorescein/glyburide pair exhibited inhibitor (glyburide)-dependence in the cellular uptake of fluorescent probe (fluorescein) in MCF-7 cells (**Fig. 2A**). The cells’ ability to exclude fluorescein (but not rhodamine 123 and mitoxanthrone) is characteristic of the MRP-type transport [17], [19]. This agrees with early reports on intrinsic drug-efflux activity in MCF-7 cells [20]–[22] and in breast cancers [23]. Thus, only MRP type of transport and its regulation were further studied in depth.

For kinetic characterization of MDR in MCF-7 cell populations, the cells were first loaded with fluorescein in the presence of glyburide and then allowed to extrude it after removal of extracellular fluorescein and glyburide by repeated centrifugation and washing. Kinetics of fluorescein efflux was derived from the mean values of the fluorescent intensity obtained from flow cytometry measurements at appropriate time intervals (**Figs. 2B, 2C**). All flow cytometry histograms acquired in this study that demonstrated a gradual expulsion of fluorescein from MCF-7 cells produced a unimodal pattern (**Fig. 2B**). This suggests that the observed fluorescence kinetics mainly reflects fluorescein transport in the bulk population of cells that have a uniform distribution of MDR activity. In this way some important aspects of intrinsic MDR transport in MCF-7 cells were established as described below.

First, we determined the relation between the observed total transmembrane flux of fluorescein and MDR transport. The strong glyburide-induced inhibition of transport, shown in **Fig. 2C**, indicates that almost the entire fluorescein flux of MCF-7 cells was driven by MDR pumps (glyburide-inhibitable transporters), as has been found in some other cell types [24], [25] Kinetic traces of fluorescein efflux could be fitted using the integrated Michaelis-Menten equation (**Fig. 2D**) which is in agreement with previously reported Michaelis behaviour of ABC transporters [26], [27]. Thus, Michaelis kinetic parameters of fluorescein efflux, *V*_max_ and *V*_max_/*K*_M_, can serve as an indicator of MDR activity and efficiency, respectively.

Second, we considered factors (other than transporter and substrate concentrations) that were potentially capable of affecting the time course of fluorescein efflux. These factors are: supply of energy (ATP) for active transport and supply of a likely co-substrate (reduced glutathione, GSH) for MRP transporters. To rule out these possibilities, we varied the medium content of ATP-producing glucose and the membrane-permeable ethyl ester of GSH (**Fig. 2C**). The results indicated that these factors did not limit the transport of fluorescein from MCF-7 cells under conditions employed. These facts further confirmed the validity of using the kinetic parameters of fluorescein efflux in these cells for characterizing MDR activity.

### Population vs Single-cell Approach in Kinetic Studies of Unimodal Cell Populations

**Figure 3:**
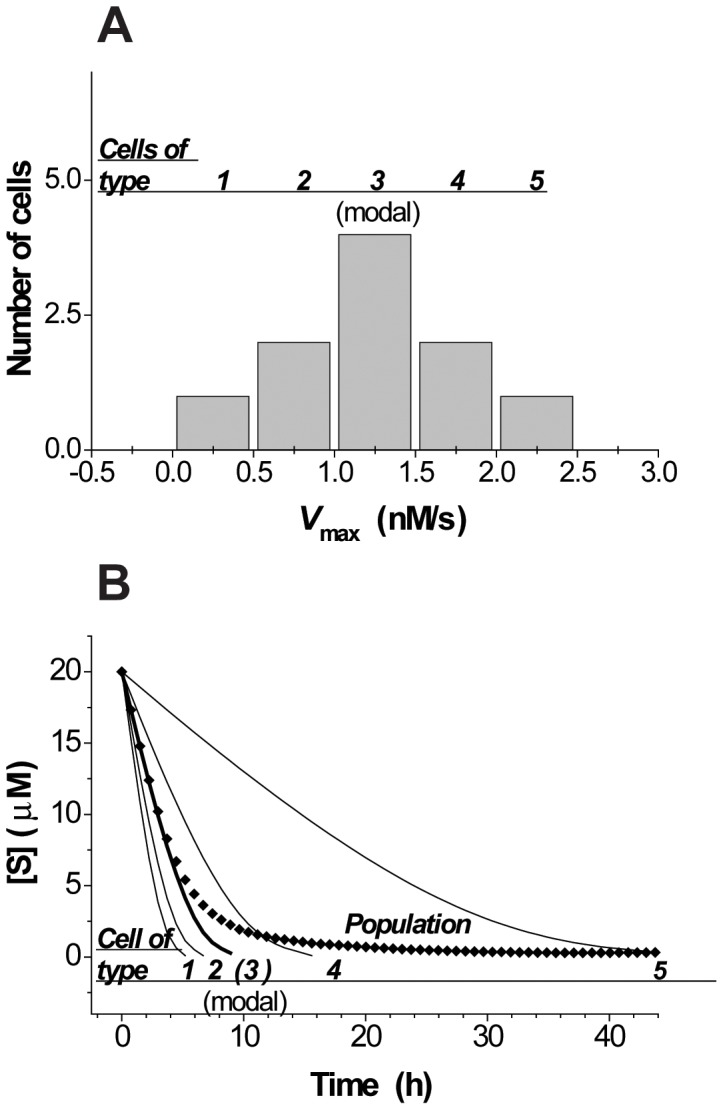
Simulation of fluorescein efflux kinetics assuming Michaelis-type process in model single cells and cell population. Simulation of single-cell and population kinetics of a Michaelis-Menten-type reaction. ***A***, variation of *V*_max_ in a population of 10 individual cell cells. ***B***, simulated single-cell and averaged population kinetics, simulation performed using the integrated Michaelis-Menten equation: *V*_max_ *t*  =  ([S]_0_ – [S]) + *K*_M_ ln([S]_0_/([S]_0_ – [S])).

Kinetics of MDR transport is typically studies using whole cell populations [25], [28], [29], an approach that is considered to be accurate in establishing the main trends of unimodal cell ensembles [30]. However, a recent work done by Wong and co-authors had shown that averaging cell kinetics over the cell population can significantly disturb reaction order determination when the cell-to-cell variation within a population is high (25–160-fold in the enzyme activity and/or substrate content) [31]. For our system, we were interested in whether or not population averaging could introduce considerable errors into measurements of Michaelis-Menten parameters at moderate (2–10-fold) cell-to-cell variation in transporter activity. To answer this question, we initially performed kinetic simulation on the cell population model. We considered a group of cells with identical *K*_M_ values and initial substrate levels but with varying *V*_max_ (distributed in a Gaussian manner over a 9-fold range) (**Fig. 3A** **,** **Table 1**). Kinetic traces of substrate extrusion for each cell were simulated using the integrated Michaelis-Menten equation (**Fig. 3B**). Kinetics of individual cells could be processed in two ways described below (see **Fig. 1**).

**Table 1:** Kinetic parameters used for simulation of single-cell kinetics and parameters derived from single-cell and population kinetics.

Sample	*n*	*V*_max_, nM/s	*K*_M_, µM	[S]_0_, µM
Cell of type 1	1	0.25	5	20
Cell of type 2	2	0.75	5	20
Cell of type 3	4	1.25	5	20
Cell of type 4	2	1.75	5	20
Cell of type 5	1	2.25	5	20
Parameters derived from averaging single-cell Michaelis parameters		1.25	5	20
Parameters derived from fitting of averaged raw trace (for 3-fold variation of *V*_max_)		2.85±0.28	24.31±2.53	20

Rows 2 to 7 show types of cells forming a model population and their kinetic parameters. Row 7 shows population *V*_max_ and *K*_M,_ obtained by averaging single-cell *V*_max_ and *K*_M_. Row 8 shows population *V*_max_ and *K*_M,_ obtained by fitting population kinetics to the integrated Michaelis-Menten equation.

Firstly, the kinetic parameters for each cell can be deduced from single-cell kinetic traces by fitting to the integrated Michaelis-Menten equation and then averaged for the population. These averages will be equal to averages of the input parameters used for the simulations (**Table 1**, row 6). In particular, the averaged *V*_max_ is equal to the *V*_max_ value of the modal cell type (**Fig. 3A**, central bin). Thus, the averaged kinetic parameters of the population coincide with the parameters of the modal cell type, which is expected for Gaussian distributions.

Secondly, instead of averaging Michaelis-Menten parameters, one can average kinetic traces (**Fig. 3B**). Note that in an experiment, this happens when the total signal from the cell population is registered by methods such as kinetic flow cytometry, spectrofluorometry, and whole-field microscopy. From **Fig. 3B** **,** one can see that averaged population kinetics generated in this way deviates significantly from the kinetics of the modal cell type (**Fig. 3B**, the thicker trace corresponds to the cells of type 3). The averaged population trace deviates from the modal population trace because the fastest cells tend to slow down and complete the reaction earlier than the slowest ones, and the further the reaction proceeds toward completion, the greater contribution slow cells have on the averaged population kinetics. For this reason, averaged population traces generated in this way have a higher curvature than the modal population traces, and lead to an erroneous perception of cellular kinetics. As a result, even though each single cell closely abides by Michaelis-Menten kinetics, for a 9-fold variation in *V*_max_, the population-averaged trace fits this kinetics very poorly. For a 3-fold variation, Michaelis fitting appears to be satisfactory visually, but produces a several-fold overestimation of *V*_max_ and *K*_M_ (**Table 1**, row 7).

Two other kinetic parameters, *K*_M_ and [S]_0_, were subjected to analogous variation at the single-cell level, and caused qualitatively similar but quantitatively smaller overestimation in their averaged population level values (20–50% of the mean parameter value at the 3-fold variation, data not shown). Such a difference might be of limited importance in the analysis of real scattered experimental data. At the same time, a several-fold overestimation of the population *V*_max_ and *K*_M,_ caused by cell-to-cell *V*_max_ variation, would create an entirely wrong picture of the process under study.

It should ne noted that both the population and single-cell approach would give identical results if the cell population analyzed were perfectly homogeneous, which is, obviously, never the case. The population approach always results in systematic errors when applied to a heterogeneous cell population and the errors increase with increasing heterogeneity. The single-cell approach is correct from the standpoint of mathematical statistics and, thus, always returns kinetic data correctly describing the cell population analyzed no matter how heterogeneous is the population. Thus, for the elucidation of potential kinetic differences between cells in different stages of the cell cycle, we applied the single-cell approach.

### Modulation of MDR Kinetics in the Course of Cells’ Progression through the Cell Cycle

The kinetics of fluorescein efflux from single cells was measured using quantitative time-lapse fluorescence confocal microscopy. Calibration of cellular fluorescence was performed in two steps. First, fluorescence from intracellular fluorescein and fluorescein in solution were compared. For this purpose, we tested the emission from the suspension of fluorescein-loaded cells before and after cell lysis with 0.1% Triton X-100. The release of the dye from the cells had no significant effect on its fluorescence, showing that the fluorescent signal from intracellular fluorescein could be quantitated using fluorescein solutions for calibration. Secondly, after each series of cell measurements, standard solutions of fluorescein were placed into the cell chamber, and their signals were recorded with the same optical settings. Linear emission-concentration dependence generated in this way (data not shown) allowed us to determine absolute concentrations of intracellular fluorescein (the focal volume was smaller than the cell volume).

**Figure 4:**
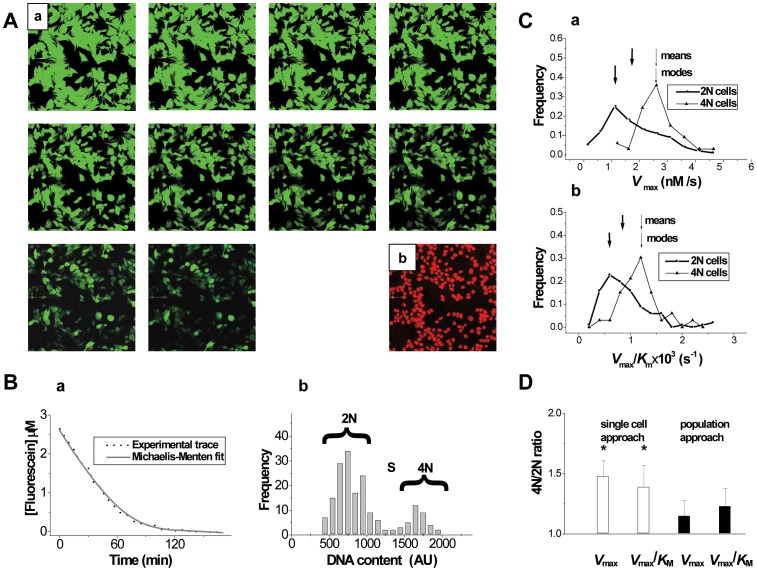
Characterization of intrinsic MDR in MCF-7 single cells in 2c and 4c states. *A*, (a) Representative fluorescence images (scan size 100×100 µm) of fluorescein efflux followed by PI staining (**b**) in MCF-7 cells used for measurements of transport kinetics and cell-cycle progression, respectively. ***B***, (**a**) typical kinetic trace showing transport of fluorescein from a single cell and its Michaelis-Menten fit, and (**b**) cell-cycle histogram obtained after completion of fluorescein transport. ***C***, frequency polygons showing characteristic distributions of *V*_max_ (**a**), and *V*_max_/*K*_M_ (**b**) values among cells in the 2c and the 4c states (typical distributions representing 4 independent cell populations are shown). Arrows show mean and modal positions in distributions of MDR parameters (thick and thin arrows correspond to 2c and 4c cells, respectively). ***D***, 4c/2c ratios of mean values of *V*_max_, and *V*_max_/*K*_M_ obtained by parallel application of single cell- (empty columns) and population-(filled columns)-oriented analysis to MDR kinetics in 2c and 4c subpopulations, representing 4 independent cell preparations.

Fluorescein efflux was initiated by removal of glyburide, which inhibits the membrane pump, and monitored until apparent completion (**Figs. 4A** **a and 4Ba**). Afterwards, the cells were treated with PI to identify apoptotic cells in order to exclude them from the quantitative analysis. Finally, by the addition of saponin (which permeates the plasma membrane) and RNAase (which removes interfering RNA), PI was allowed to enter all cells and stain the DNA (**Fig. 4A** **b**). Fluorescence intensity of PI was used to assign cells to either 2c (G1/G0) phase or 4c (G2/M) phase (**Fig. 4B** **b**), so that the kinetics of dye efflux from single cells belonging to the first and second halves of the cell cycle (before and after DNA duplication) could be estimated

Fluorescein efflux from the majority of single MCF-7 cells conformed to Michaelis-Menten kinetics, with the exception of approx. 5% of cells that had demonstrated PI staining before the permeation and/or abnormal efflux kinetics; these cells were excluded from analysis. **Figure 4C** shows that cell-cycle transition from the 2c phase to the 4c phase was accompanied with a shift in the MDR activity (characterized by *V*_max_) and efficiency (characterized by *V*_max_/*K*_M_) distributions toward the higher grades. Accordingly, the 4N cells demonstrated elevated mean values of *V*_max_ and *V*_max_/*K*_M_ (upper arrows in **Fig. 4C** frequency polygons, and statistical summary in **Fig. 4D**). This difference was even greater in terms of modal levels of MDR in 4c and 2c subpopulations (lower arrows in **Fig. 4C** frequency polygons). The modal values of MDR activity characterize the most abundant cell type in the population and often determines tumor behaviour [32]. Here, we did not consider the likely presence of a side population with elevated MDR activity (*V*_max_), which could exert only a minor contribution to the overall distribution of MDR activity within the entire population.

To compare the resolving power of the population and single-cell kinetics approaches, we carried out a parallel analysis of the same raw data using both methods. **Figure 4D** shows MDR kinetic parameters in the 4c and 2c states, determined with the two approaches. The single-cell derived 4c/2c ratios of *V*_max_ and *V*_max_/*K*_M_ were higher than those obtained from the population averaging calculations, and only the single-cell values were significantly different from unity, which suggests a higher resolving power for the single-cell approach.

## Discussion

The multitude of drug-selected drug-resistant cell types widely used in MDR studies tend to acquire a greater-than physiological level of resistance, and are likely to be regulated in variable fashions [33], making their relevance to MDR functioning in vivo widely debated [34], [35]. Therefore, in this work, we studied the physiologically occurring intrinsic multidrug resistance, which determines drug selection and outcome for the first round of chemotherapy.

The majority of cellular processes are modulated by the cell’s progression through the cell cycle [36], and one can expect that the intrinsic activity of MDR transport in cancer cells is not an exception. Few studies, in which this issue was addressed, considered the expression of specific ABC transporters in different phases of the cell cycle [2], [4], [5]. At the same time, it was noticed that widely-used measurements of MDR gene expression in tumor samples were not always clinically effective because they did not necessarily provide information on the functional activity of drug efflux pumps [6], [37]. Controversial clinical results on the correlation between chemoresistance and the expression of ABC transporters [38], [39] are likely to reflect the contribution of factors, other than transporter expression, in determining MDR activity. In particular, the progression of cells through the cell cycle is accompanied by alterations in many parameters (cellular energetic state, physical state of plasma membrane, and levels of cofactors), that can potentially affect the kinetic parameters of MDR transporters. We assumed that the intensity of drug efflux is the most relevant indicator of a cells chemoresistance capacity and reflects both the MDR pump expression and molecular catalytic activity in their entirety. The detected here MDR transport of MRP-type only in MCF-7 cells agrees with intrinsic expression of MRP1 proteins and a lack of MDR1 proteins reported earlier (24).

A few functionally-oriented studies were previously performed that had employed an indirect, semiquantitative assessment of MDR efficiency and suggested the activation of MDR transport in S and G2/M phases in leukemic cells [40]. Here we applied a rigorous quantitative methodology to studying cell cycle-related MDR modulation.

During the last decade, a neoadjuvant therapeutic approach, aiming at a reduction in tumor mass as to facilitate further treatment, is finding increasing use. In light of this approach, the chemoresistance of the main population of tumor cells along with that of the tumor-initiating cells, are of crucial importance. Thus, in this work we studied the whole population of MCF-7 cells.

The application of single-cell approach to 2c and 4c subpopulations of clonal breast cancer cells revealed an increase in the mean values of both MDR activity and efficiency in the 4N cells; this provides the first quantitative kinetic evidence for changes in MDR transport during the cell-cycle progression. MDR activation in the 4N cells was even more pronounced when characterized by modal (instead of mean) values of MDR parameters within subpopulations **(** **Fig. 4C** **)**. The characterization of MDR in the most abundant cell type in a tumor would be important in the prognosis of the tumor’s immediate response to chemotherapy, which plays basal role in neoadjuvant therapeutic approach. Investigation of the stem-like subpopulation of cancer cells will be required to understand if the increase in long-term efficiency of cell-cycle selective chemotherapy can be expected.

The advantages of the single-cell kinetics approach for studies of the unimodal cell populations used in our experiments, was tested by comparing results from single-cell and population (**Fig. 1**) analyses of the same data sets (**Fig. 4D**). The population kinetic parameters (*V*_max_ and *V*_max_/*K*_M_) obtained from the single-cell approach grew significantly upon cells’ transition from the 2c to the 4c state. At the same time, these parameters obtained from the population approach remained almost unchanged upon such a transition.

Most tumors are believed to originate from a single cell but multiple clones are typically selected during tumorigenesis and perceived at the advanced stages. Hence, real tumor tissues are expected to be more heterogeneous than a single cultured clone. As a result, the population approach, which gives errors for a single cultured clone, will lead to even greater errors for real tumors. Accordingly, the benefit of using the single-cell approach will be even greater if real tumor tissues are analysed.

When the single-cell approach is applied to real tumor tissues, the analyzed cell population may include cells from different clones. While the single-cell approach will correctly determine kinetic parameters describing the analyzed population, the interpretation of the results should always be done carefully. For example, if the single-cell approach reveals that MDR efflux correlates with the cell cycle for a mixture of clones, we can conclude with great certainty that all clones behave in a similar way with respect to MDR efflux. However, if no correlation is found, two interpretations are possible: (i) MDR does not correlated with the cell cycle or (ii) MDR correlates differently for different clones and the different correlations cancel each other when a mixture of clones is pulled together. To find the right answer, one would need to conduct extensive additional studies on MDR kinetics in individual clones. We think that comparing different cultured clones should be the next step in an attempt to find out if different clones within the same tumor vary with respect to MDR-cell cycle correlation. While being very cumbersome with an ordinary microscope, such a study could be perfectly feasible if an automated image cytometry is used.

The experimentally-found wide variation of Michaelis-Menten parameters in single cells is consistent with a few reports in which single-cell Michaelis parameters were dealt with [41]. One of the reasons for this variation could be the existence of two forms of the transporter, with each having unique properties [42], [43].

It was shown that many cell types arrested in the cell cycle [44], including those arrested in the G2 phase [3], [45], can demonstrate an elevated resistance to anticancer treatment which is usually explained by the modulation of apoptotic and mitotic mechanisms. The concept of cell cycle-mediated drug resistance derived from these studies aims at understanding and optimizing cell cycle-based drug interactions [3]. Our data suggests that the variation in multidrug transport activity can strongly contribute to the cell-cycle-related modulation of chemoresistance.

Potential translation of our experimental approach to clinical practice will require further extensive work, in particular, studying relations between particular ABC transporters and cytostatic agents. Since one can expect that the heterogeneity of tumor tissues is higher than that of cell lines, the single-cell approach would be especially beneficial in this case.

The presence and activity of MDR transporters in clinical specimens is considered to be a valuable prognostic indicator for many forms of cancer [8], [46]. A clinical version of our single-cell kinetics assay may further strengthen the evaluation of MDR in tumor biopsies and make this evaluation more quantitative.
